# *RASSF1A* and *DOK1* Promoter Methylation Levels in Hepatocellular Carcinoma, Cirrhotic and Non-Cirrhotic Liver, and Correlation with Liver Cancer in Brazilian Patients

**DOI:** 10.1371/journal.pone.0153796

**Published:** 2016-04-14

**Authors:** Oscar C. Araújo, Agatha S. Rosa, Arlete Fernandes, Christian Niel, Cristiane A. Villela-Nogueira, Vera Pannain, Natalia M. Araujo

**Affiliations:** 1 Laboratory of Molecular Virology, Oswaldo Cruz Institute, FIOCRUZ, Rio de Janeiro, Brazil; 2 Department of Pathology, Clementino Fraga Filho University Hospital, Federal University of Rio de Janeiro, Rio de Janeiro, Brazil; 3 Hepatology Division, Clementino Fraga Filho University Hospital, Federal University of Rio de Janeiro, Rio de Janeiro, Brazil; CRCL-INSERM, FRANCE

## Abstract

Hepatocellular carcinoma (HCC) is the second most common cause of cancer mortality worldwide. Most cases of HCC are associated with cirrhosis related to chronic hepatitis B virus or hepatitis C virus infections. Hypermethylation of promoter regions is the main epigenetic mechanism of gene silencing and has been involved in HCC development. The aim of this study was to determine whether aberrant methylation of *RASSF1A* and *DOK1* gene promoters is associated with the progression of liver disease in Brazilian patients. Methylation levels were measured by pyrosequencing in 41 (20 HCC, 9 cirrhotic, and 12 non-cirrhotic) liver tissue samples. Mean rates of methylation in *RASSF1A* and *DOK1* were 16.2% and 12.0% in non-cirrhotic, 26.1% and 19.6% in cirrhotic, and 59.1% and 56.0% in HCC tissues, respectively, showing a gradual increase according to the progression of the disease, with significantly higher levels in tumor tissues. In addition, hypermethylation of *RASSF1A* and *DOK1* was found in the vast majority (88%) of the HCC cases. Interestingly, *DOK1* methylation levels in HCC samples were significantly higher in the group of younger (<40 years) patients, and higher in moderately differentiated than in poorly differentiated tumors (*p* < 0.05). Our results reinforce the hypothesis that hypermethylation of *RASSF1A* and *DOK1* contributes to hepatocarcinogenesis and is associated to clinicopathological characteristics. *RASSF1A* and *DOK1* promoter hypermethylation may be a valuable biomarker for early diagnosis of HCC and a potential molecular target for epigenetic-based therapy.

## Introduction

Liver cancer is the second leading cause of cancer-related mortality, with an estimated 700,000 deaths each year worldwide [1]. Hepatocellular carcinoma (HCC) is by far the most common type of primary liver cancer and one of the few cancers with well-defined major risk factors. Approximately 80% of all HCC cases are associated with chronic hepatitis B virus (HBV) or hepatitis C virus (HCV) infections [2], although chronic alcoholism and nonalcoholic steatohepatitis (NASH) are also major causes [3, 4]. These risk factors induce chronic liver damage often leading to cirrhosis, which is present in 80–90% of patients with HCC [4, 5]. Since HCC prognosis depends on tumor stage at diagnosis, lower survival rates have been observed among patients at the advanced stage [6], highlighting the importance of the identification of biomarkers for early liver cancer detection and therapeutic interventions.

The molecular mechanisms underlying hepatocarcinogenesis remain unclear. Similar to other tumors, the development and progression of HCC are due to a multistep process including accumulation of genetic and epigenetic alterations in regulatory genes, which lead to the activation of different oncogenes and inactivation of tumor suppressor genes. Hypermethylation of promoter CpG islands is an epigenetic mechanism of gene silencing involved in a wide range of human cancers, including HCC [7, 8]. Aberrant DNA methylation has also been demonstrated in premalignant conditions such as dysplastic nodules or cirrhotic liver [9–11], suggesting that it is an early event in hepatocarcinogenesis and a valuable marker for risk assessment. The Ras association domain family 1A (*RASSF1A*) and downstream of tyrosine kinases 1 (*DOK1*) are tumor suppressor genes frequently silenced through epigenetic mechanism in a variety of human malignancies [12, 13]. Aberrant methylation of these genes has been proposed to play an important role in the malignant transformation of the hepatocyte [10, 14]. Notably, hypermethylation of these genes in HCC has been reported to show geographical variations, suggesting that ethnicity may influence DNA methylation levels [10].

Brazilian people are the result of an intense admixture between European colonizers or immigrants, African slaves, and native Amerindians. In Brazil, almost 9,000 deaths were attributed to HCC in 2013 [15], and chronic HCV infection is the major risk factor, accounting for more than 50% of the cases [16]. To our knowledge, no report has been published so far on the epigenetic factors associated to HCC in Brazil.

In this study, we performed a quantitative DNA methylation analysis by pyrosequencing in Brazilian patients at HCC, cirrhotic, and non-cirrhotic stages, and assessed the relationship between methylation levels and clinicopathological characteristics, with the aim of examining the relevance of the hypermethylation of *RASSF1A* and *DOK1* as a molecular marker of HCC.

## Materials and Methods

### Ethics Statement

The study protocol was approved by the Research Ethics Committee of the Clementino Fraga Filho University Hospital (approval number 139/10) and performed in accordance with the declaration of Helsinki. Due to the retrospective nature of the study, the Ethics Committee concluded that no written informed consent was required from the patients.

### Patients and tissue samples

Archived formalin-fixed paraffin-embedded tissue blocks were obtained at the Department of Pathology of the Clementino Fraga Filho University Hospital, Rio de Janeiro, Brazil. Liver samples from 20 patients with HCC, four with cirrhosis without HCC, and 12 with chronic hepatitis (non-cirrhotic) were analyzed (Table 1). Additionally, the surrounding cirrhotic tissue was analyzed for five HCC patients (total = 41 samples). Histological analysis revealed features compatible with chronic hepatitis (inflammation with or without fibrosis) in all non-cirrhotic liver tissues. HCC and cirrhotic tissue samples were obtained from liver explants or surgical resection, while tissues from patients with chronic hepatitis were obtained by percutaneous liver biopsy. All HCC patients included in this study had cirrhotic livers.

**Table 1:** Clinicopathological characteristics of the patients.

	HCC (n = 20)	Cirrhosis only^a^ (n = 4)	Chronic hepatitis only^b^ (n = 12)	Total (n = 36)
Age (years)				
<40	3 (15%)	0	1 (8%)	4 (11%)
40–60	7 (35%)	1 (25%)	5 (42%)	13 (36%)
≥60	10 (50%)	3 (75%)	4 (33%)	17 (47%)
ND	0	0	2 (17%)	2 (6%)
Gender				
Male	16 (80%)	4 (100%)	6 (50%)	26 (72%)
Female	4 (20%)	0	4 (33%)	8 (22%)
ND	0	0	2 (17%)	2 (6%)
Viral status^c^				
HBV	5 (25%)	0	2 (17%)	7 (19%)
HCV	10 (50%)	1 (25%)	9 (75%)	20 (56%)
No virus	2 (10%)	3 (75%)	1 (8%)	6 (17%)
ND	3 (15%)	0	0	3 (8%)
Tumor differentiation				
Well	0	NA	NA	NA
Moderately	14 (70%)			
Poorly	6 (30%)			
Tumor size				
<5 cm	8 (40%)	NA	NA	NA
>5 cm	10 (50%)			
ND	2 (10%)			
Tissue samples (n = 41)				
HCC	20	0	0	20
Cirrhotic	5	4	0	9
Non-cirrhotic	0	0	12	12

^a^Without HCC;

^b^Without HCC or cirrhosis;

^c^HBV, HBsAg positive; HCV, anti-HCV and HCV-RNA positive; No virus, HBsAg, anti-HCV and HCV-RNA negative; NA, not applicable; ND, not determined.

Demographic and clinicopathological data were obtained from medical records in order to evaluate the association of age, gender, histology, and HBV and HCV infections with DNA methylation. The viral status of the patients was established as follows: HBV infection, HBsAg positive; HCV infection, anti-HCV and HCV-RNA positive; no virus, HBsAg, anti-HCV and HCV-RNA negative.

The mean age of the patients was 57 (range 19–78) years, with 47% of them aged ≥ 60 years. Twenty-six (72%) patients were men. HCV infection was the main etiological factor of liver disease (56% of the cases), followed by HBV (19%). Fourteen (70%) HCC patients had a moderately differentiated tumor, and ten (50%) had a tumor size >5 cm (Table 1).

### DNA isolation and sodium bisulfite conversion

Five sections of 10 μm were cut from each block, deparaffinized in 1 mL of absolute xylene, and rehydrated in 1 mL of 96% ethanol. Genomic DNA extraction was carried out by using the QIAamp DNA FFPE Tissue Kit (Qiagen, Valencia, CA), according to the manufacturer’s protocol. Approximately 500 ng of genomic DNA was subjected to bisulfite modification treatment to convert all unmethylated cytosines to thymines. This was performed by using the EZ DNA Methylation-Lightning Kit (Zymo Research, Irvine, CA). The modified DNA was stored at -20°C for further analysis.

### DNA methylation analysis by pyrosequencing

DNA methylation levels of *RASSF1A* and *DOK1* promoter regions were measured by the highly sensitive pyrosequencing technology (PyroMark Q96 ID, Qiagen) at multiple CpG sites. Bisulfite-treated DNA was PCR amplified in a volume of 50 μL reaction, which contained 1U of Platinum Taq DNA Polymerase, High Fidelity (Invitrogen, Carlsbad, CA) and 0.2 μM of each oligonucleotide primer [10], under the following conditions: 94°C for 30 sec; 35 cycles at 94°C for 30 sec, 55°C for 30 sec, and 68°C for 1 min, followed by a final elongation step at 68°C for 7 min. Ten microliters of PCR product was analyzed on agarose gel by electrophoresis. The remaining 40 μL of the biotinylated PCR product was captured on streptavidin-coated beads (GE Healthcare, Milwaukee, WI) and the pyrosequencing reaction was set up using the PyroMark Gold Q96 kit (Qiagen), according to the manufacturer’s instructions. The set of sequencing primers has been previously designed [10]. The percentages of methylation were measured at six and five CpG sites in the *RASSF1A* and *DOK1* promoters, respectively, and expressed as the means of all CpGs analyzed at a given gene. To assess DNA hypermethylation frequencies in HCC and cirrhotic samples, cut-off values for *RASSF1A* and *DOK1* were obtained from the quantile representing the upper 95% of methylation levels in non-cirrhotic samples, as previously defined [10]. Therefore, the frequencies of DNA hypermethylation were represented as the percentage of HCC and cirrhotic samples with methylation levels above the cut-off value for each gene.

### Statistical analysis

All statistical analyses were performed using the SPSS 20.0 software (IBM, Armonk, NY). The Kruskal-Wallis test (or Mann-Whitney U test, when appropriate) was used to compare methylation levels in HCC, cirrhotic, and non-cirrhotic liver samples, as well as to test associations between methylation levels and clinicopathological characteristics. Type 1 error was adjusted for multiple comparisons. Differences were considered statistically significant for *p* values less than 0.05.

## Results

### DNA methylation levels in non-cirrhotic, cirrhotic and HCC tissue samples

**Fig 1:**
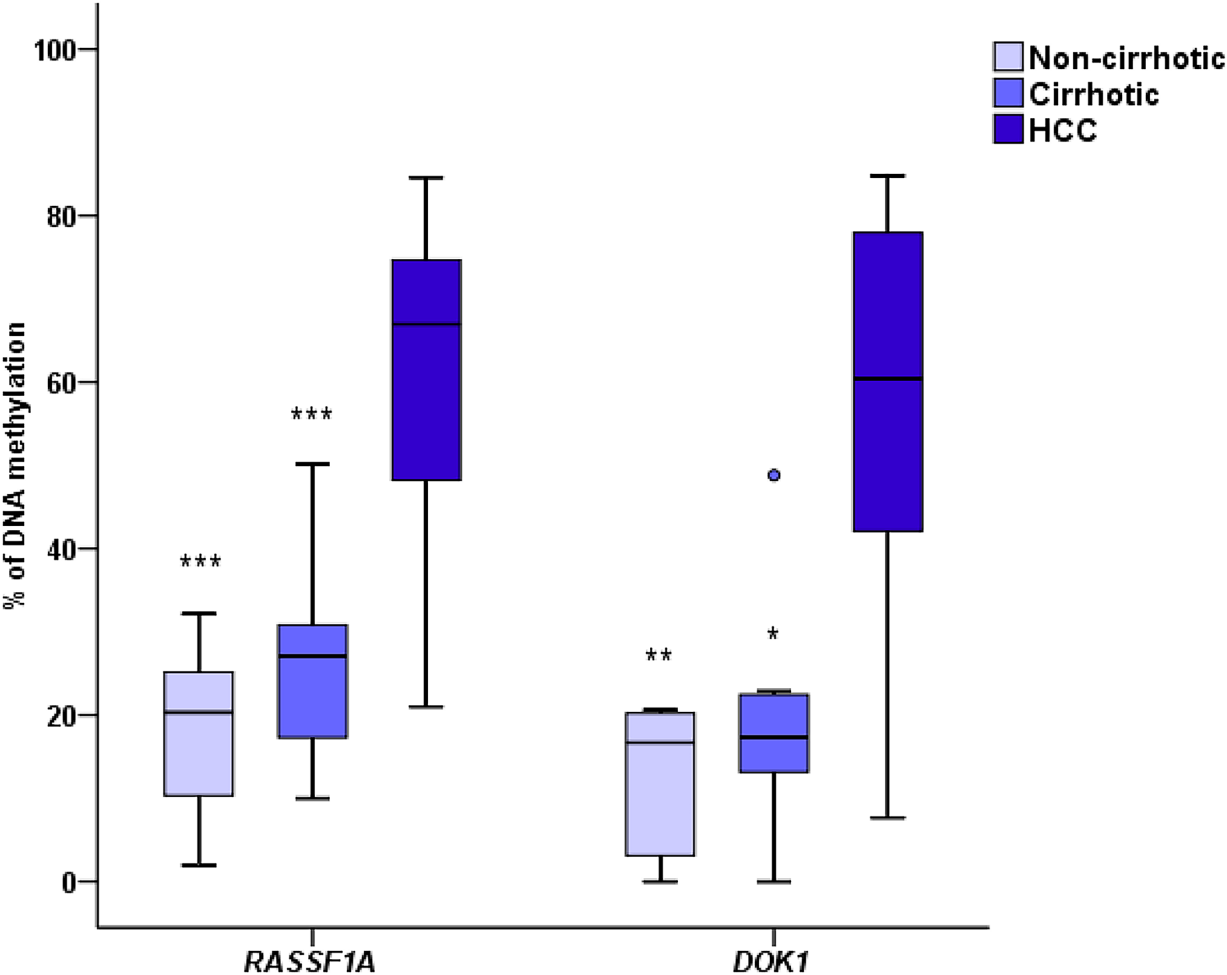
Boxplot showing *RASSF1A* and *DOK1* promoter methylation levels in non-cirrhotic, cirrhotic, and HCC tissues. Methylation levels were measured by pyrosequencing at different CpG sites and expressed as the mean percentage of all CpGs analyzed at a given gene. The statistical significance for DNA methylation level in non-cirrhotic and cirrhotic tissues was calculated by comparison to HCC samples.

Pyrosequencing analysis was successfully performed in 41 (20 HCC, 9 cirrhotic and 12 non-cirrhotic) and 31 (17 HCC, 7 cirrhotic and 7 non-cirrhotic) tissue samples for *RASSF1A* and *DOK1*, respectively. The results of the quantitative analysis of DNA methylation, expressed as the mean percentage of all CpGs analyzed at a given gene, are shown in Fig 1. Overall, methylation levels were lower in non-cirrhotic, moderate in cirrhotic, and higher in HCC tissues for both genes. Mean rates of methylation in *RASSF1A* and *DOK1* were as follows: 16.2% and 12.0% in non-cirrhotic, 26.1% and 19.6% in cirrhotic, and 59.1% and 56.0% in HCC tissues, respectively (Table 2). No statistical difference in methylation levels was detected between cirrhotic and non-cirrhotic tissues (*RASSF1A*, *p* = 0.165; *DOK1*, *p* = 1.000). However, we observed a highly significant difference of methylation levels between HCC and non-HCC tissues for both genes (Table 2). Methylation levels in cirrhotic tissues from patients without HCC and adjacent cirrhotic tissues from patients with HCC were also compared. The differences between these two groups were not statistically significant (*RASSF1A*, *p* = 0.652; *DOK1*, *p* = 0.231). A correlation of *DOK1* methylation levels was observed between HCC- and cirrhotic paired samples, with HCC/cirrhotic paired tissues displaying simultaneously the highest or the lowest levels of methylation. However, such a correlation was not observed for *RASSF1A* methylation (data not shown).

**Table 2:** Correlation between tissue type and DNA methylation levels.

				*p* values		
Gene	Non-cirrhotic	Cirrhotic	HCC	Cirrhotic x Non-cirrhotic	HCC x Non-cirrhotic	HCC x Cirrhotic
*RASSF1A*, mean ± SD (%)	16.2 ± 9.4	26.1 ± 11.7	59.1 ± 19.3	0.165	< 0.001	< 0.001
*DOK1*, mean ± SD (%)	12.0 ± 9.6	19.6 ± 15.0	56.0 ± 23.3	1.000	0.003	0.012

SD, standard deviation

### DNA methylation status and clinicopathological parameters

The associations between clinicopathological characteristics (age, gender, and histology) and risk factors (HBV and HCV infections) on one hand, and methylation levels in HCC tissues on the other hand, are shown in Table 3. Overall, *RASSF1A* and *DOK1* exhibited similar methylation patterns according to the parameters analyzed. We found a statistically significant difference in *DOK1* methylation levels from an age group to another (*p* = 0.036), with higher methylation levels in the group of younger (<40 years) patients. In addition, significantly higher levels (*p* = 0.005) of *DOK1* methylation were observed in moderately differentiated- than in poorly differentiated HCCs. Although *RASSF1A* and *DOK1* methylation levels were higher in women and in HBV positive tumors, no statistical difference could be observed. Also, no association was found between tumor size and methylation levels (Table 3).

**Table 3:** DNA methylation levels in HCC samples stratified by clinicopathological characteristics and viral status.

	*RASSF1A*		*DOK1*	
	mean (%)	*p* value	mean (%)	*p* value
Age				
<40	61.7	0.945	74.8	0.036
40–60	57.1		36.9	
≥60	59.7		60.3	
Gender				
Men	56.9	0.395	51.2	0.059
Women	68.0		78.1	
Viral status				
HBV	58.5	0.792	74.7	0.227
HCV	56.2		53.7	
No virus	54.9		42.8	
Tumor differentiation				
Moderately	62.4	0.284	68.0	0.005
Poorly	51.4		34.0	
Tumor size				
<5 cm	55.4	0.374	56.2	0.540
>5 cm	61.9		57.9	

### DNA hypermethylation frequencies in cirrhotic and HCC tissues

Analysis of the DNA hypermethylation frequencies revealed that *RASSF1A* and *DOK1* genes were hypermethylated in the vast majority (15/17, 88%) of the HCC tissues (Table 4). Moreover, the simultaneous hypermethylation of both genes was detected in 14/17 (82%) HCC tissues. However, lower frequencies of hypermethylation were observed among cirrhotic tissues (2/7, 29% in *RASSF1A*; 3/7, 43% *DOK1*; and 2/7, 29% both genes). With respect to viral status, all HBV positive HCCs showed hypermethylation in *DOK1*. The concomitant hypermethylation of these genes was more frequently observed in HBV (3/4, 75%) and HCV (8/9, 89%) positive tumors than HBV and HCV negative ones (1/2, 50%). Similarly, concurrent hypermethylation was detected in 2/3 (67%) HCV positive cirrhotic tissues, but in none (0/4) of the HCV negative ones (Table 4). Frequencies of hypermethylation were similar between cirrhotic tissues from patients without HCC and adjacent cirrhotic tissues from patients with HCC (data not shown).

**Table 4:** Frequency of DNA hypermethylation according to tissue type and viral status.

	HCC^a^				Cirrhotic^a^				
Gene	All^b^^,^^c^ (n = 17)	HBV (n = 4)	HCV (n = 9)	No virus (n = 2)	All^b^ (n = 7)	HBV (n = 0)	HCV (n = 3)	No virus (n = 4)	Cut-off
*RASSF1A*	15 (88%)	3 (75%)	8 (89%)	2 (100%)	2 (29%)	NA	2 (67%)	0	32.15%
*DOK1*	15 (88%)	4 (100%)	8 (89%)	1 (50%)	3 (43%)	NA	2 (67%)	1 (25%)	20.62%
Both	14 (82%)	3 (75%)	8 (89%)	1 (50%)	2 (29%)	NA	2 (67%)	0	-

^a^Samples with methylation levels above the quantile representing the upper 95% of methylation in non-cirrhotic samples.

^b^Only samples having both *RASSF1A* and *DOK1* methylation levels were included in the analysis.

^c^Viral status is not available for two samples.

NA, not applicable.

## Discussion

In the present study, we have analyzed quantitatively the methylation profile of *RASSF1A* and *DOK1* promoter genes in non-cirrhotic and cirrhotic livers without HCC, as well as in HCC and cirrhotic paired samples. Both genes have tumor suppressor properties and are involved in several key cellular processes. *RASSF1A* is implicated in the Ras signaling pathway, which plays a pivotal role in cell cycle control, microtubule stabilization, cellular adhesion, cell motility, and apoptosis [17]. *DOK1* is expressed in B- and T cells as well as in myeloid cells (macrophages and neutrophils), and is involved in a wide range of immunoreceptor signaling pathways [18]. Herein, *RASSF1A* and *DOK1* methylation levels showed a gradual increase according to the progression of the disease, with significantly higher levels in tumor tissues. Additionally, we observed very high rates of *RASSF1A* (88%) and *DOK1* (88%) hypermethylation in HCC samples, with a concomitant hypermethylation of 82%. Previous studies using different methodologies, as well as different sets of CpGs, have also found high (76–100%) frequencies of *RASSF1A* hypermethylation in HCC [10, 19–21]. On the other hand, few data are available on the methylation profile of *DOK1* in liver cancer. One study reported the occurrence of hypermethylation in the majority (62%) of the HCC tissues [10]. Our results thus reinforce the hypothesis of an association between epigenetic silencing of these key cellular genes and hepatocarcinogenesis. Of note, we found concomitant *RASSF1A* and *DOK1* hypermethylation in almost 30% of the cirrhotic tissues analyzed. Aberrant methylation was found not only in cirrhotic samples of patients with HCC, but also in those without HCC, suggesting that the increase of methylation levels in a subset of cirrhotic tissues due to the presence of some tumor cells is unlikely. These findings are in keeping with the notion that aberrant methylation in non-transformed hepatocytes may precede their oncogenic transformation and promote the development of liver cancer.

The potential influence of ethnicity in DNA methylation has not been extensively studied. However, previous reports have described significant differences in global genomic DNA methylation according to ethnicity [22, 23]. It has been suggested that some genetic polymorphisms, such as those of folate metabolizing enzymes, may contribute to the ethnic differences in DNA methylation [24]. Lambert et al. [10] have shown an association between methylation levels in several genes and geographical area, with HCCs exhibiting *RASSF1A* and *DOK1* methylation levels lower in Thailand than in France. Our study relied on a set of CpGs analyzed previously, and measured DNA methylation levels by the quantitative pyrosequencing methodology [10]. Interestingly, we observed higher *RASSF1A* and *DOK1* methylation levels in Brazilian HCC samples as compared to those from Thailand (2.7 and 3.1 fold) and France (1.9 and 1.8 fold), respectively. These results corroborate the notion that genetic and/or environmental factors may contribute to the ethnic/geographical differences of DNA methylation among HCC cases.

Several studies have investigated potential associations between risk factors (HBV and HCV infections) or clinical features (age, gender, and histology) and DNA methylation in HCC [9, 10, 19–21, 25]. Promoter-associated CpG island methylation has been considered one of the most prominent molecular changes that can be observed in aging [26]. Noteworthy, a previous study has shown that all patients over 40 years had the *RASSF1A* promoter methylated in non-neoplastic cells of the liver [27]. However, we observed higher levels of *RASSF1A* and *DOK1* methylation in HCC tissues of the younger group (<40 years), with a statistically significant difference between *DOK1* methylation and age (*p* = 0.036). Herein, all HCC patients under 40 years of age were chronically infected with HBV, and one may suppose that these patients were infected a long time ago, probably through vertical transmission. Several studies have shown that the HBV X protein (HBx) increases total DNA methyltransferase (DNMT) activities by upregulation of DNMTs, promoting hypermethylation of specific tumor suppressor genes [28]. Therefore, the viral status of the youngest patients may have contributed to the higher methylation levels observed in our study. Nevertheless, previous studies have also found higher levels of *RASSF1A* and *DOK1* methylation in HCC samples in the younger groups, although the differences were not statistically significant [9, 10]. In addition, significantly higher levels of *DOK1* methylation in cirrhotic tissues in the age group <40 years have been previously reported [10]. Finally, we found that levels of *RASSF1A* and *DOK1* methylation were higher in moderately differentiated- than in poorly differentiated HCCs, with a significant difference in *DOK1* (*p* = 0.005). None of the HCC patients included in our study had well differentiated tumors, so that a better comparison between DNA methylation and tumor grade of differentiation could not be achieved. In a previous work, *RASSF1A* methylation was almost equal regardless of HCC differentiation [9], whereas there are no reports about *DOK1* methylation and HCC grade of differentiation to date.

In conclusion, this is the first report on the epigenetic factors associated to HCC in Brazil. Our results confirm that hypermethylation of *RASSF1A* and *DOK1* contributes to hepatocarcinogenesis and is an early event in cancer development, likely associated to clinical characteristics as well as major risk factors. Therefore, *RASSF1A* and *DOK1* aberrant methylation may be a valuable biomarker for early diagnosis of HCC and an attractive molecular target for epigenetic-based therapy. Non-invasive approaches, such as those measuring hypermethylation of the circulating cell-free DNA in serum/plasma, should be encouraged.
